# Genome-Wide Co-Expression Analysis in Multiple Tissues

**DOI:** 10.1371/journal.pone.0004033

**Published:** 2008-12-29

**Authors:** Ian C. Grieve, Nicholas J. Dickens, Michal Pravenec, Vladimir Kren, Norbert Hubner, Stuart A. Cook, Timothy J. Aitman, Enrico Petretto, Jonathan Mangion

**Affiliations:** 1 MRC Clinical Sciences Centre, Imperial College, Hammersmith Hospital, London, United Kingdom; 2 Institute of Cancer Research, Belmont, Sutton, Surrey, United Kingdom; 3 Institute of Biology and Medical Genetics, First Faculty of Medicine and General Teaching Hospital, Charles University, Prague, Czech Republic; 4 Institute of Physiology, Czech Academy of Sciences, Prague, Czech Republic; 5 Max-Delbrűck-Center for Molecular Medicine, Berlin-Buch, Berlin, Germany; 6 National Heart and Lung Institute, Imperial College, London, United Kingdom; 7 Division of Epidemiology, Public Health and Primary Care, Imperial College, London, United Kingdom; University of Cape Town, South Africa

## Abstract

Expression quantitative trait loci (eQTLs) represent genetic control points of gene expression, and can be categorized as *cis*- and *trans*-acting, reflecting local and distant regulation of gene expression respectively. Although there is evidence of co-regulation within clusters of *trans*-eQTLs, the extent of co-expression patterns and their relationship with the genotypes at eQTLs are not fully understood. We have mapped thousands of *cis*- and *trans*-eQTLs in four tissues (fat, kidney, adrenal and left ventricle) in a large panel of rat recombinant inbred (RI) strains. Here we investigate the genome-wide correlation structure in expression levels of eQTL transcripts and underlying genotypes to elucidate the nature of co-regulation within *cis*- and *trans*-eQTL datasets. Across the four tissues, we consistently found statistically significant correlations of *cis*-regulated gene expression to be rare (<0.9% of all pairs tested). Most (>80%) of the observed significant correlations of *cis*-regulated gene expression are explained by correlation of the underlying genotypes. In comparison, co-expression of *trans*-regulated gene expression is more common, with significant correlation ranging from 2.9%–14.9% of all pairs of *trans*-eQTL transcripts. We observed a total of 81 *trans*-eQTL clusters (hot-spots), defined as consisting of ≥10 eQTLs linked to a common region, with very high levels of correlation between *trans*-regulated transcripts (77.2–90.2%). Moreover, functional analysis of large *trans*-eQTL clusters (≥30 eQTLs) revealed significant functional enrichment among genes comprising 80% of the large clusters. The results of this genome-wide co-expression study show the effects of the eQTL genotypes on the observed patterns of correlation, and suggest that functional relatedness between genes underlying *trans*-eQTLs is reflected in the degree of co-expression observed in *trans*-eQTL clusters. Our results demonstrate the power of an integrative, systematic approach to the analysis of a large gene expression dataset to uncover underlying structure, and inform future eQTL studies.

## Introduction

The use of linkage analysis in combination with genome-wide expression profiling by microarray, also known as ‘genetical genomics’ [1], enables the genetic control points of gene expression to be mapped to the genome. These have come to be referred to as expression quantitative trait loci (eQTLs) [2]. This study design has in the past five years been applied to the investigation of regulatory processes in, among others, yeast, rodents, plants and humans [3], [4], [5], [6], [7].

A major strength of the eQTL approach is the inherent ability to distinguish between local and distant regulation [8] of gene expression, by classifying eQTLs as *cis* and *trans*. *Cis*-eQTLs are those in which the eQTL maps to the physical location of the transcript [5]. *Cis*-eQTLs have been shown generally to be highly heritable and to have a larger genetic effect than *trans*-eQTLs [9]. Of particular interest are those *cis*-eQTLs that co-localise with mapped physiological quantitative trait loci (pQTLs) [5], [10], [11], [12], as these can be considered to be strong candidates for the genetic regulation underlying variation in the physiological trait. To prioritize *cis*-eQTLs, Schadt *et al* applied data resulting from the correlation of expression profiles with phenotypic measurements to identify candidate genes underlying physiological QTLs in mice [10]. Similar procedures have been carried out in other model organisms [7], [11], [13], [14], [15]. Using these approaches in humans, Deutsch *et al* [16] and Goring *et al* [17] identified candidate genes for Down's syndrome phenotypes and plasma HDL cholesterol concentration respectively.

*Trans*-eQTLs are those in which the expression of a given gene maps to a location remote from the physical location of the gene itself. *Trans*-eQTLs are considered indicative of gene expression levels that are under polygenic control [9]. Because *trans*-eQTLs generally have small genetic effects and often a correspondingly high False Discovery Rate (FDR), they are relatively difficult to detect [9], [17]. They are however of interest as potential indirect regulators of gene expression [18]. It has been observed that *trans*-eQTLs form clusters, or ‘hot-spots’ [19], where mRNA levels of transcripts across the genome show linkage to the same genetic locus. Such *trans*-eQTL clusters have also been detected through the application of quite straightforward correlation-based methods [19], [20] or functionally-informed correlation analysis [7]. The possibility of functional enrichment within *trans*-eQTL clusters has been explored in other datasets [20], [21], with outcomes suggesting that the presence of clusters is predictive of biological relationships between the underlying genes.

Co-expressed, functionally enriched *trans*-eQTL clusters are suggestive of co-ordinated regulation, perhaps by a ‘master’ transcriptional regulator located within the linkage region [3], [22]. Previous attempts to prioritise candidate regulators *in silico* have involved the correlation of the expression levels of the genes comprising the *trans*-eQTL cluster with those of candidate regulators co-localising with the cluster locus [18].

Correlation-based methods have been applied to prioritise candidate genes or to infer co-expression networks, but in many cases genotype information is not utilised in the analysis [23], [24]. Others have taken into account the effect of correlation due to linkage disequilibrium [12], [25] but not long-range allelic association [26].

Here, we extend these analyses, taking into account the correlation of the genotypes underlying *cis*- and *trans*-eQTLs in multiple tissues. We carried out a full-scale integrative co-expression analysis of gene expression in a large panel of rat recombinant inbred (RI) strains, the BXH/HXB panel [27]. Genotypic and genetic map information was fully integrated into the analysis. We show that taking into account genetic distance data is requisite in the prediction of candidate regulators of *trans*-eQTL clusters. The outcomes of this study provide new insight into the relationship between gene expression and underlying genotype in *cis*- and *trans*-eQTLs across multiple tissues.

## Results

### Correlation of gene expression levels of cis- and trans-acting eQTLs

**Figure 1:**
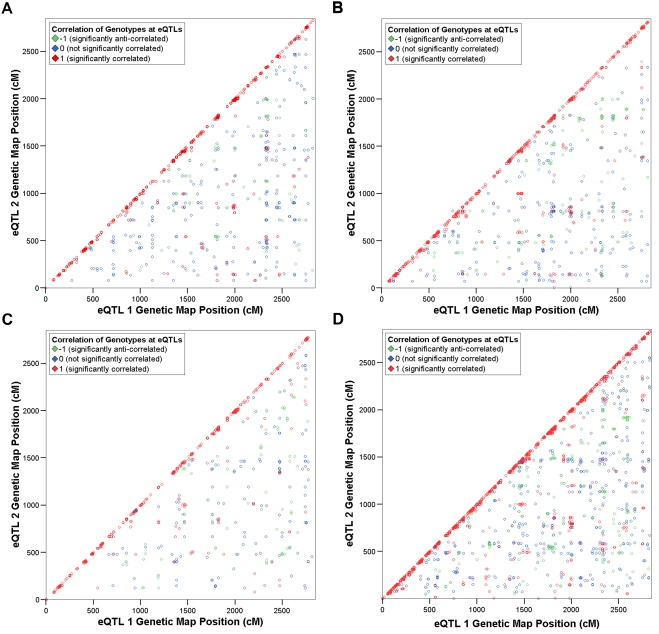
Scatter plots showing pairs of *cis*-eQTL genes with significantly correlated expression profiles by genetic map location. Significantly correlated pairs of *cis*-eQTL genes identified in a) fat, b) kidney, c) adrenal and d) left ventricle are plotted according to genetic map location (cM), indicating significance or otherwise of correlation of strain distribution patterns (SDPs) at the peak of linkage. Colour coding indicates significance of correlation of SDPs (*p*<0.05; see Methods). Pairs consisting of probesets located close to one another, which correspondingly have nearby peaks of linkage, are disproportionately represented among significantly correlated pairs as indicated by the red diagonal.

Pairwise correlation analysis was performed in all eQTLs detected with genome-wide significance *p*<0.05 in fat, kidney, adrenal and left ventricle tissues (Table 1). The correlation structure in genes that form *cis*-eQTLs were consistently found to differ markedly from that in genes that form *trans*-eQTLs in all tissues. Gene expression levels of *cis*-eQTLs were found to be significantly (FDR<0.05) correlated to one another in 0.6 to 0.9% of all pairs (Table 2). 80.4 to 91.5% of these were found to have correlated genotypes across the RI strains (i.e., Strain Distribution Patterns (SDPs)), most of which (>67.9% (Table S1)) could be explained by linkage disequilibrium (LD) (Figure 1). Of the significantly correlated pairs of *cis*-eQTLs that can not be explained by LD, 39.6 to 55.6% have correlated SDPs (Table S1), even though the eQTLs are often located on different chromosomes. The remainder of the correlated pairs of *cis*-eQTL genes cannot be explained by similarity of the underlying genotypes.

**Table 1:** Numbers of *cis*- and *trans*- expression QTLs identified in each of the four rat tissues at genome-wide corrected *p*≤0.05.

Tissue	No. of *cis*-eQTLs*	No. of *trans*-eQTLs*
Fat	558	923
Kidney	718	1,033
Adrenal	602	933
Left Ventricle (LV)	1,362	2,140
Total	3,240	5,029

* *cis*- and *trans*-eQTLs are defined as follows: A *cis*-eQTL is one in which the peak of linkage is within 10 Mb of the transcript. Any other eQTL is defined as a *trans*-eQTL.

**Table 2:** Outcomes of correlation analysis of *cis*-eQTL genes in each of the four tissues.

Tissue	Total Number of Pairs of *cis*-eQTLs	No. Significantly Correlated Pairs of *cis*-eQTL genes^a^ (*q*<0.05)	% Significantly Correlated Pairs of *cis*-eQTL genes^a^	No. (%) Correlated Pairs^a^ with Significantly Correlated Genotypes^b^ at Peaks of Linkage
Fat	155,403	1,386	0.9	1,114 (80.4%)
Kidney	257,403	2,284	0.9	1,979 (86.6%)
Adrenal	180,901	1,443	0.8	1,294 (89.7%)
LV	926,841	5,371	0.6	4,912 (91.5%)

a Significantly correlated pairs are defined as those in which the absolute Pearson correlation of the gene expression levels was empirically found to be statistically significant (FDR<0.05), following control for multiple testing.

b Correlation of genotypes at a pair of eQTLs across the RI strain panel was assessed by calculating the matching coefficient. Genotypes are described as significantly correlated if the total number of matched pairs is found to be significant by a two-tailed test of probability (*p*<0.05).

**Figure 2 pone-0004033-g002:**
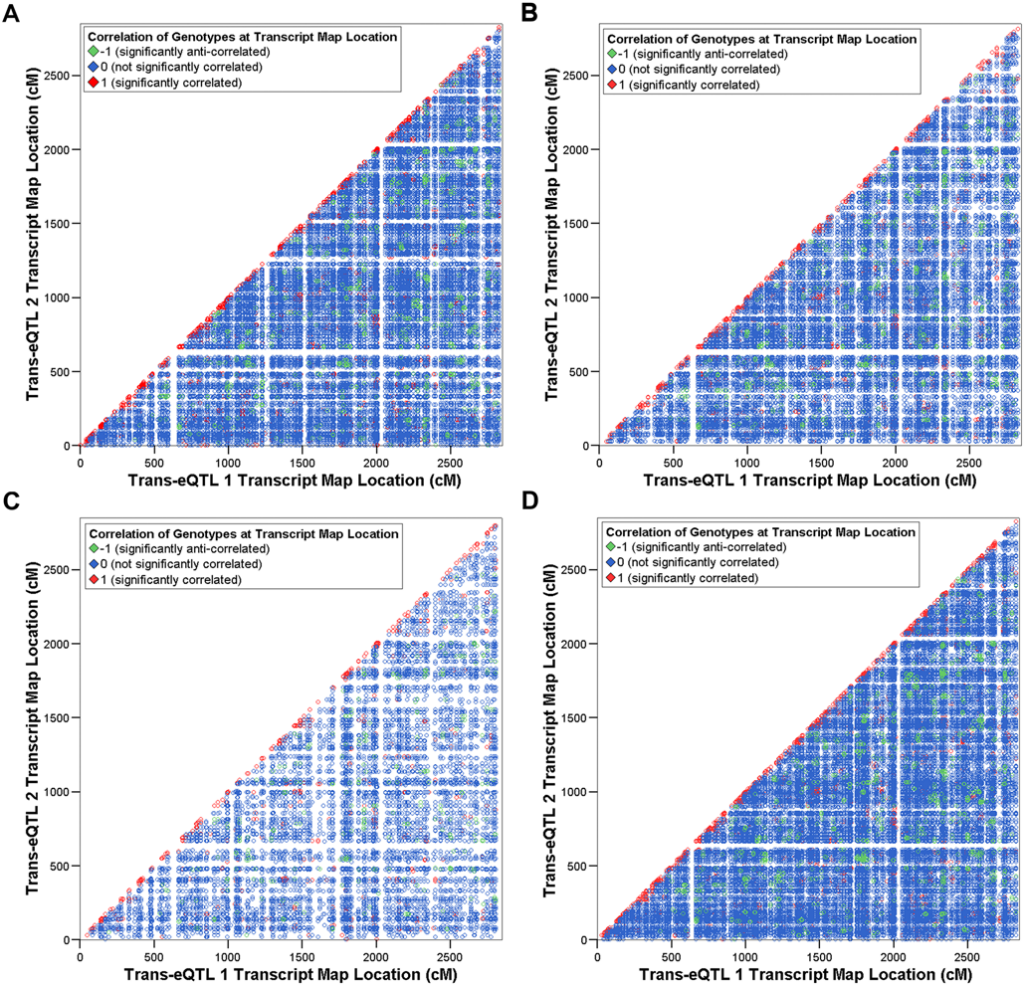
Scatter plots showing pairs of *trans*-eQTL genes with significantly correlated expression profiles by transcript genetic map location. Significantly correlated pairs of *trans*-eQTL genes identified in a) fat, b) kidney, c) adrenal and d) left ventricle are plotted according to genetic map location of the transcripts. Colour coding is as in Figure 1.

**Figure 3:**
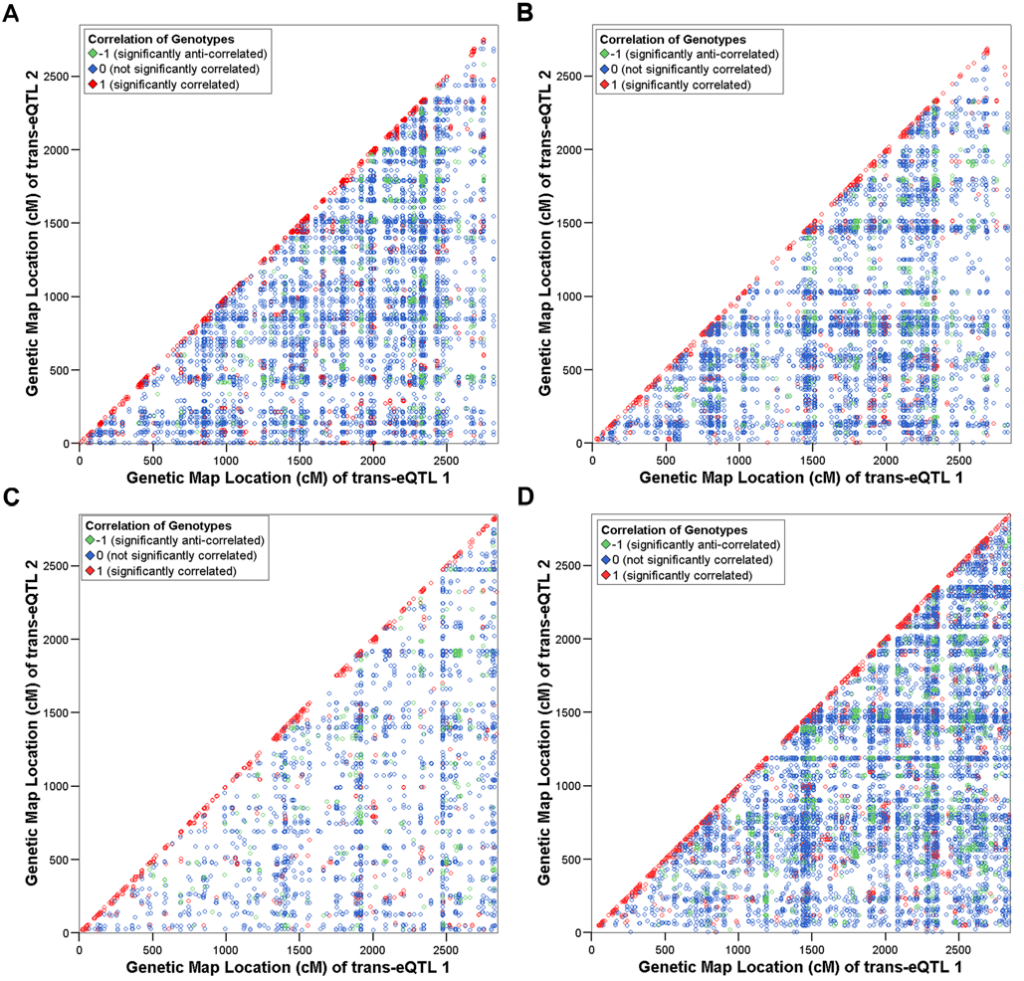
Scatter plots showing pairs of *trans*-eQTL genes with significantly correlated expression profiles by genetic map location of peak of linkage. Significantly correlated pairs of *trans*-eQTLs identified in a) fat, b) kidney, c) adrenal and d) left ventricle are plotted according to genetic map position of the peak of linkage. Colour coding is as in Figure 1. Over-representation of pairs of *trans*-eQTLs with nearby peaks of linkage among the significantly correlated set can be observed (in the red diagonal) in all four tissues.

Consistently across all tissues, we found a greater proportion of pairs of *trans*-eQTL genes (2.9 to 14.9%) that showed significant correlation of expression levels (Table 3), compared with *cis*-eQTLs. Unlike *cis*-eQTLs, a substantial overrepresentation of pairs of *trans*-eQTLs whose underlying genes are both located on the same chromosome was not observed (Figure 2). However, when the SDPs at the significantly correlated *trans*-eQTLs were tested for correlation (Figure 3), we detected a high proportion (47.5 to 63.7%) of significantly (*p*<0.05) similar SDPs (Table 3). This was investigated more closely by analysing the distances between the map locations of these *trans*-eQTLs. We found that 23.1 to 39.1% of significantly correlated *trans*-eQTL genes form eQTLs that are located within 1 cM of one another (Table S2). This suggests the presence of groups, or clusters, of co-regulated *trans*-eQTLs.

**Table 3:** Outcomes of correlation analysis of *trans*-eQTL genes in each of the four tissues.

Tissue	Total Number of Pairs of *trans*-eQTLs	No. Significantly Correlated Pairs of *trans*-eQTL genes^a^ (*q*<0.05)	% Significantly Correlated Pairs of *trans*-eQTL genes^a^	No. (%) Correlated Pairs^a^ with Significantly Correlated Genotypes^b^ at Peaks of Linkage	No. (%) Correlated Pairs^a^ with Significantly Correlated Genotypes^b^ at Probeset Locations
Fat	425,503	63,477	14.9	31,288 (49.3%)	6,375 (10.0%)
Kidney	533,028	39,257	7.4	18,628 (47.5%)	4,601 (11.7%)
Adrenal	434,778	12,482	2.9	7,945 (63.7%)	1,370 (11.0%)
LV	2,288,730	78,826	3.4	44,110 (56.0%)	8,450 (10.7%)

a b As described in Table 2.

### Co-regulation analysis of trans-eQTL clusters

**Figure 4:**
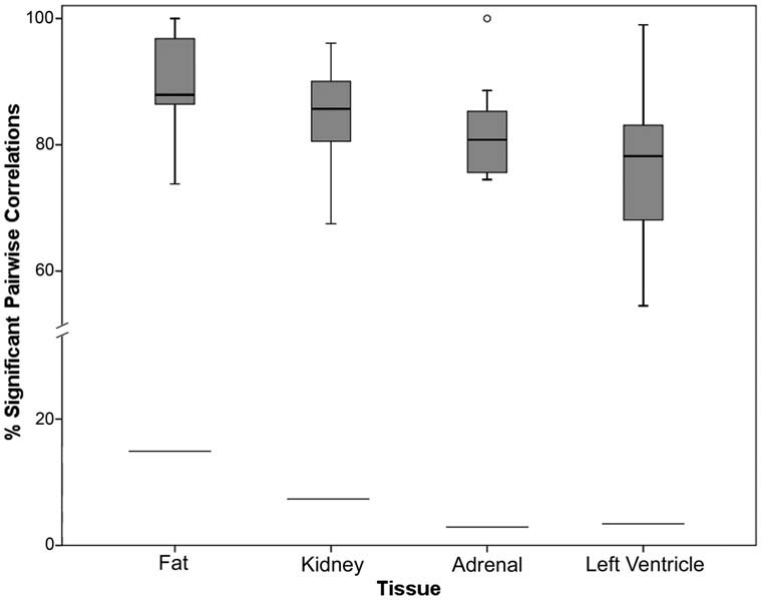
Boxplots showing percentage within-cluster correlation of genes underlying *trans*-eQTLs forming *trans*-eQTL clusters. For each of the four tissues, the percentages of significantly correlated pairs of *trans*-eQTL genes within *trans*-eQTL clusters are displayed. The boxplots indicate the median, interquartile range, and range of within-cluster correlation in each tissue. The percentage significant correlation among all trans-eQTLs is shown for each of the four tissues as horizontal lines, for purpose of comparison with the *trans*-eQTL clusters. One outlier is shown, a cluster in adrenal found to have 100% significant pairwise correlation of gene expression levels (see Table S3).

A total of 81 *trans*-eQTL clusters, defined as 10 or more co-localising *trans*-eQTLs, were identified across all four tissues (Table S3). Levels of significant correlation within *trans*-eQTL clusters (Figure 4) were found to be much greater than in the rest of the *trans*-eQTL dataset. Significant correlations ranged from 77.2 to 90.2% of all pairs (Table 4) whereas in the *trans*-eQTL dataset as a whole they ranged from 2.9 to 14.0% of all pairs (Table 3).

**Table 4:** Summary of outcomes of pairwise correlation of *trans*-eQTL cluster genes.

Tissue	No. of *Trans*-eQTL Clusters (≥10 *trans*-eQTLs)	Mean *Trans*-eQTL Cluster Size (no. of *trans*-eQTLs)	*Trans*-eQTL Cluster Ave. % Significantly Correlated Pairs^a^
Fat	11	30.8	90.2
Kidney	23	19.5	84.2
Adrenal	9	19.5	82.4
LV	38	25.1	77.2
**Overall**	**81**	**23.7**	**83.5**

a As described in Table 2.

We tested for functional enrichment within *trans*-eQTL clusters through Gene Ontology (GO) annotations using DAVID, a web-based functional annotation tool [28]. This analysis was performed on 15 large *trans*-eQTL clusters consisting of 30 or more transcripts, because smaller clusters were considered unlikely to provide sufficient statistical power for such an analysis (see Methods). Of these, 12 (80%) were found to have at least one significant biological process or metabolic function GO term at an uncorrected significance level of *p*<0.01 and almost half have at least one significant term at uncorrected *p*<0.001 (Table 5). To assess the significance of these findings, simulation studies were carried out using DAVID on 200 random sets of 47 unique *trans*-eQTL genes. We found a significantly increased functional enrichment at all three GO *p*-value thresholds (*p*<0.001, *p*<0.01, *p*<0.05) in the *trans*-eQTL datasets relative to the random sets (Kolmogorov-Smirnov Exact test *p*-values: *p* = 0.001, *p* = 0.002, *p* = 0.004).

**Table 5:** Outcome of GO analysis of *trans*-eQTL clusters consisting of 30 or more eQTLs using DAVID, a functional annotation software tool.

Tissue	Marker at Cluster Peak of Linkage	No. Cluster *trans*-eQTLs	Significant GO terms at *p*<0.001	Significant GO terms at *p*<0.01	Significant GO terms at *p*<0.05
LV	D15Rat98	30	0	0	1
Adrenal	D11Rat16	31	0	2	7
Fat	D4Rat240	31	2	4	8
Fat	Cacna1s	33	0	1	4
LV	Cyp45c	35	0	0	0
LV	Ckb	43	3	4	12
LV	D15Ucsf1	46	0	2	12
Adrenal	D17Rat144	47	0	1	8
Kidney	Igk	49	1	3	14
LV	D15Utr2	51	2	13	20
LV	D15Rat29	54	8	15	28
Kidney	D15Rat69	57	9	13	20
LV	D8Mit12	77	0	0	3
Fat	D17Rat1	146	3	20	53
LV	Crabp1	165	0	4	14

### Prediction of candidate ‘master regulators’

**Figure 5:**
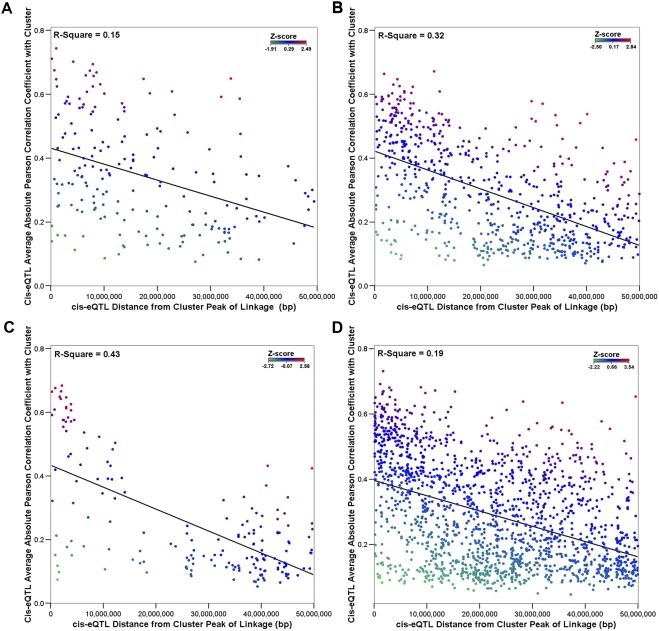
Scatter plots showing correlation of genes underlying cluster *trans*-eQTLs and *cis*-eQTLs located in the window region, plotted against their distance from the peak of linkage. Each point on the scatter plot represents a *cis*-eQTL within the defined window region of a *trans*-eQTL cluster. The average Pearson coefficient of correlation of the underlying transcript expression levels of the *cis*-eQTL with those of the cluster *trans*-eQTLs is shown to be strongly negatively correlated with distance in a) fat, b) kidney, c) adrenal, d) LV, R^2^ for this relationship ranges from 0.15 to 0.43. The Z-score, calculated from the vertical distance of each *cis*-eQTL from the regression line, is indicated by the colour of each point, indicated by the colour legend in the top right of each plot.

In order to prioritize *cis*-eQTLs as candidate ‘master regulators’ [19] of the *trans*-eQTL clusters, expression profiles of all *cis*-eQTL genes located within 50 Mb either side of the cluster locus were tested for co-expression with those of the *trans*-eQTL cluster genes. We found levels of correlation to be dependent on the distance of the *cis*-eQTL from the cluster peak of linkage. Upon linear regression of correlation against distance between gene and peak of linkage, *R^2^* in all four tissues was found across clusters to show a negative relationship (Figure 5) with distance from the peak; ranging from 0.15 to 0.43.

The analysis was subsequently carried out on all transcripts located in the same region, regardless of whether they form eQTLs or not. Regression analysis between distance and average correlation with the *trans*-eQTL cluster genes showed no relationship (R^2^: 0–0.01) (Figure S1), indicating that the relationship observed in *cis*-eQTLs is explained by the linkage of those probesets to the genetic region concerned. In order to investigate the extent of genetic control of gene expression in the *cis*-eQTLs in the region, we also calculated transcript heritability as previously described [9]. There was found to be no relationship between distance of the *cis*-eQTL from the cluster linkage region and heritability of the *cis*-eQTL (Figure S2).

These findings suggest that correlation of expression levels of *cis*-eQTLs in the vicinity of the peak of linkage of a *trans*-eQTL cluster with those of the *trans*-eQTLs making up said cluster, without taking into account factors relating to genetic linkage is inappropriate for the purpose of identifying putative regulators. Analysis of outliers of the relationship between distance and correlation may hold more promise to identify candidates than simply looking at highly correlated eQTLs. For this purpose, Z-scores (based on the outcome of the regression analysis) were calculated for each tissue. *Cis*-eQTLs with significant Z-scores (defined as Z>2) in each tissue are shown in Table S5. These *cis*-eQTLs may represent candidates for the regulation of the *trans*-eQTL clusters.

## Discussion

Our genome-wide studies of expression levels of genes underlying *cis*- and *trans*-eQTLs provide strong support for the hypotheses that the categorisation of eQTLs has a genuine biological basis that can be detected in transcript expression levels, and that *trans*-eQTL clusters consist of functionally related and co-ordinately regulated transcripts.

We observed that patterns of correlation of expression profiles and of SDPs at the genetic location of the transcript whose expression was measured are strikingly different between the sets of *cis*- and *trans*-eQTLs in all four tissues (Figures 1–3). Significant correlation between pairs of *cis*-eQTLs was found to be rare (<1%, Table 2). A significant relationship has previously been shown between the absolute correlation coefficient of pairs of eQTLs derived from genes located on the same chromosome and the distance apart of those genes [10], [24]. Hence it was hypothesised that correlation of *cis*-eQTL genes can be explained in terms of linkage disequilibrium. We found that most of the observed correlations between *cis*-eQTL genes' expression profiles for co-localised genes can be explained by similarity of underlying genotypes. We also found that most significant correlations between *cis*-eQTLs located on different chromosomes can be explained by long-range allelic association. Such patterns of association have previously been observed in a SNP map of a mouse inbred strain panel [26], and hypothesised to have the potential to produce spurious associations between transcripts' expression profiles. Our findings suggest that correlation of expression profiles, in isolation, is not a suitable basis for the analysis of relationships between *cis*-eQTLs.

Much higher levels of significant correlation, encompassing 2.9–14.9% of pairs, were observed between *trans*-eQTL genes. This was not found to be explained by correlation of the genotypes at the regions to which the transcripts map. However, when genotypes at the *trans*-eQTL are similar or the same, the gene pairs were disproportionately correlated (47.5 to 63.7% of significantly correlated pairs). This observation provides strong support for the hypothesis, formulated in studies of genetic regulation of gene expression in *S. cerevisiae* [19], that *trans*-eQTL clusters are indicative of co-regulation of the transcripts. In our dataset, the level of significant correlation within *trans*-eQTL clusters was found to be far higher than across the *trans*-eQTL dataset as a whole, averaging 83.5% across the 81 clusters (Table 4). Additionally, we observed that much of the variability in the levels of *trans*-eQTL correlation between tissues can be explained by differences in the distribution and size of the *trans*-eQTL clusters (data not shown).

Functional investigation of the large *trans*-eQTL clusters showed significant over-representation of Gene Ontology (GO) terms in 80% of these clusters. While GO analysis may have limitations in scope and accuracy, owing to the annotation challenges posed by genes which often have complex and imprecisely defined roles and functions [29], the findings presented here are in agreement with those of Ghazalpour *et al* [25], who observed that genes in functionally related ‘pathway sets’ are typically highly correlated. KEGG pathway analysis of the *trans*-eQTL clusters was carried out through the DAVID interface, and pathways of potential interest were identified in a minority of the clusters (Table S6). It has previously been shown in a study of co-regulation in a mouse F2 intercross [20] that groups of highly correlated transcripts linked to the same genomic location can be identified in a functionally informed genome-wide correlation analysis. Here we show across multiple tissues that highly correlated expression profiles are a consistent motif of such *trans*-eQTL clusters. These results suggest a significant degree of functional relatedness of the genes making up the cluster (Table 5).

Expression correlation between *trans*-eQTL cluster transcripts and *cis*-eQTL genes located in the linkage region has been previously described as a method of identifying candidate regulators and applied in yeast [30] and *Arabidopsis* [7]. Here we show a strong relationship between the distance of the *cis*-eQTL from the cluster peak of linkage and the strength of correlation (Figure 5), which was not observed when unlinked transcripts located in the region were similarly tested (Figure S1). This, along with the observation that there is no association between distance from the peak of linkage and heritability of the *cis*-eQTL genes (Figure S2), suggests that genotype similarity at the linkage region, rather than the genetic influence on gene expression underlies the relationship. On this basis, correlation-based methods of prioritising candidates for genetic regulation of *trans*-eQTL clusters would be improved by taking into account the distance between the cluster linkage region and the map location of the candidate transcript. We therefore suggest that it may be of interest instead to identify outliers; those *cis*-eQTL genes whose average correlation with the *trans*-eQTL cluster genes significantly deviates positively from the general negative regression trend. We were able to find 54 *cis*-eQTLs with significant positive Z-scores (Z>2) (Table S4), and consider that these may be worthy of further investigation into the possibility that their correlation with the cluster transcripts has a biological basis.

In this study, we demonstrate the power of computational analysis of eQTL datasets across multiple tissues to provide new insights into the genome-wide correlation structure in gene expression data. Our findings consistently show that correlation of *cis*-eQTL genes' expression profiles is primarily indicative of similarity of genetic inheritance, as measured by the correlation of SDPs at the transcript location. Whereas, correlation between *trans*-eQTLs can frequently be explained in terms of co-regulation by a common linkage region even though correlated transcripts forming *trans*-eQTL clusters are located throughout the genome. The observation of functional enrichment within clusters is suggestive of a relationship between co-expression and function. Finally, we inform investigations of candidate regulators of *trans*-eQTL clusters by indicating that genetic linkage strongly influences co-expression of *trans*-eQTL cluster genes and candidate regulatory genes.

## Materials and Methods

### The eQTL dataset

The gene expression dataset used in this study derives from the BXH/HXB panel of 29 RI strains produced from progenitor strains SHR and BN.Lx previously as described [5], [9]. Gene expression was measured in four tissues using Affymetrix GeneChips with appropriate biological and technical replication. For the retroperitoneal fat, kidney and adrenal gland tissues, Rat Expression Array 230A GeneChips were used, on which 15,923 transcripts are represented. Rat Genome 230 2.0 GeneChips, on which 31,099 transcripts are represented (including the same 15,923 as on 230A), were used for left ventricle. The summary value for each expression trait (as described in [5]) was used in the generation of the eQTLs upon which this analysis was performed. This was achieved through the application of a genome-wide linkage methodology, testing for linkage between each expression trait and the same 1,011 microsatellite markers in each of the four study tissues. QTL Reaper *(* http://www.genenetwork.org/qtlreaper.html *)* was used to carry out this analysis. The software calculates a likelihood ratio statistic (LRS) for each combination of marker and probeset, and uses permutation to estimate an empirical genome-wide probability of obtaining such a score, accounting for multiple testing [5]. The eQTLs used in this analysis are significant at genome-wide *p*<0.05.

### *Cis*- and *trans*-eQTLs

The categorisation of eQTLs as *cis* or *trans* was determined, following the merging of linkages to tightly linked markers, by eQTL Explorer [31] (http://web.bioinformatics.ic.ac.uk/eqtlexplorer). *Cis*-eQTLs were defined empirically as having a peak of linkage within 10 Mb of the physical location of the probeset as described previously [9]. Those eQTLs mapped to locations that are 10 Mb or more apart, or where the probeset is located on a different chromosome to the peak of linkage are defined as *trans*-eQTLs. Probeset location data is obtained from Affymetrix NetAffx (http://www.affymetrix.com/analysis/index.affx) and checked using EnsEMBL (http://www.ensembl.org/) (v42) data. Probesets that map to more than one place in the genome according to the latter were removed from the analysis using SCAMPA (http://microarray.csc.mrc.ac.uk/scampa/), as were those that do not map at all – in which case the *cis/trans* definition may be inaccurate. The resultant filtered set used in the correlation analysis consists of a total of 8,269 eQTLs across the four tissues, 3,240 of which are defined as *cis*-eQTLs and the remaining 5,029 as *trans*-eQTLs.

### *Trans*-eQTL clusters

A ‘*trans*-eQTL cluster’ was defined as 10 or more co-localising *trans*-eQTLs. *Trans*-eQTL clusters within 1 Mb of one another were merged following initial detection of co-ordinated linkage of at least 10 transcripts to the region. For the purpose of prioritising ‘master regulator’ candidates, the ‘window region’ of a *trans* cluster was conservatively defined as the region of the genome 50 Mb either side of the physical map location of the peak of linkage of the cluster prior to any merging.

### Correlation of Expression Data

Correlation of expression levels of genes underlying eQTLs was carried out using Matlab. A raw Pearson coefficient and associated nominal *p*-value were calculated for each pair of eQTLs. Additionally, an empirical *p*-value was determined using a permutation-based method robust against non-normality of the underlying distribution [32]. In each tissue, all pair combinations of *cis*-eQTLs and of *trans*-eQTLs were correlated. Separately, all pairs of *trans*-eQTLs within each of the 81 *trans*-eQTL clusters were correlated with each other and with the *cis*-eQTLs in the 50 Mb ‘window region’. All of these analyses entailed the testing of substantial numbers of simultaneous hypotheses. Statistical significance of obtained correlation coefficients, taking this into account, was assessed using the *q*-value (http://faculty.washington.edu/jstorey/qvalue/) [33] method of estimating the False Discovery Rate (FDR), and defined as *q*<0.05.

### Correlation Analysis of Marker SDPs

The correlation of the SDPs of the markers at the peaks of linkage of the eQTLs in each correlated pair was assessed using a matching coefficient. For each strain, a match in the marker genotypes was scored 1, and a mismatch scored 0. Strains with missing genotype data at the marker locus were not used in the calculation of the matching coefficient, which was assessed by dividing the total number of matches by the total number of strains with no missing genotypes. Because both correlation and anti-correlation of SDPs can explain significant correlation of eQTL transcript expression levels, the two-tailed significance (*p*<0.05) of the resulting coefficient was found by assessing the probability of the occurrence of all combinations, given the distribution of expected matches.

### Assessment of Functional Coherence of *Trans*-eQTL clusters

The possibility of functional enrichment in the genes that make up the observed *trans*-eQTL clusters was assessed through testing of their GO annotations using the functional annotation clustering component of the web-based genetic data analysis tool DAVID (http://david.abcc.ncifcrf.gov) [28], [34]. This analysis was performed on 15 clusters consisting of 30 or more eQTLs, since only these clusters provide enough statistical power considering that not all transcripts are annotated. Pathway analysis was performed through the same interface, using data from KEGG (http://www.genome.jp/kegg) [35]. 200 control groups of 47 probesets (47 being the median size of the 15 large *trans*-eQTL clusters) were randomly generated from the set of all *trans*-eQTLs across all four tissues and each was tested for enrichment of gene ontology terms in the same way as were the real clusters using DAVID. A Kolmogorov-Smirnov test was used to test the statistical significance of the degree of enrichment found in the 15 large *trans*-eQTL clusters, compared to the control groups. This analysis was carried out using SPSS v14.

### Distance Modelling

In the investigation of the correlation of expression levels of *trans*-eQTL clusters and colocalising *cis*-eQTLs, the relationship between correlation coefficient and distance of eQTL from the peak of linkage of the *trans*-eQTL cluster was tested by performing a regression analysis as follows: For each cluster, the genetic distance between the peak of linkage and each of the *cis*-eQTLs in the window region was found. The distance was plotted against the averaged absolute correlation coefficient of the expression profile measured by that probeset and those of the *trans*-eQTLs making up the cluster. The data for all of the clusters in each tissue was then combined to produce the plots shown in Figure 5, and linear regression was performed on the data. In addition, the relationship between heritability data (as described in Petretto *et al* [9]) and *cis*-eQTL distance from the peak of linkage was tested (Figure S2). Z-scores were calculated from the vertical distance of each *cis*-eQTL from the regression line, following testing for normality of the distribution of vertical distances. For comparative purposes, the analysis as described was also performed using all of the probesets in the ‘window region’ (as opposed to just *cis*-eQTLs) (Figure S1).

## Supporting Information

Figure S1 Scatter plots showing correlation of genes underlying cluster *trans*-eQTLs with all probesets mapped to within 50 Mb of the linkage region, plotted against the physical distance of the probeset from the peak of linkage in a) fat, b) kidney, c) adrenal, d) LV.
(6.79 MB TIF)

Figure S2 Z-score of *cis*-eQTL distance from regression line (*Figure 5*) plotted against probeset heritability in a) fat, b) kidney, c) adrenal or d) LV.
(3.24 MB TIF)

Table S1 Outcomes of correlation analysis of pairs of *cis*-eQTL genes (*q*<0.05), whose peaks of linkage are located more than 50 cM apart.
(0.03 MB DOC)

Table S2 Outcomes of correlation analysis of *trans*-eQTL genes (*q*<0.05) whose peaks of linkage are located 50 cM or less apart.
(0.05 MB DOC)

Table S3 Outcomes of correlation analysis of expression profiles of genes forming *trans*-eQTL clusters
(0.14 MB DOC)

Table S4 Outcomes of correlation of *cis*-eQTL genes in the ‘window regions’ of *trans*-eQTL clusters with cluster-forming genes.
(0.14 MB DOC)

Table S5 *cis*-eQTL genes located within the window region of a *trans*-eQTL cluster positively deviating (Z>2) from regression of cluster-averaged correlation coefficient against distance of *cis*-eQTL from linkage region (*Figure 5*).
(0.08 MB DOC)

Table S6 Supplementary information on functional enrichment analysis of large (>30 transcripts) *trans*-eQTL clusters.
(0.04 MB DOC)
