# Supplementary figures and images for: Genome-Wide Co-Expression Analysis in Multiple Tissues
Source: PLoS One. 2008 Dec 29;3(12):e4033. doi: 10.1371/journal.pone.0004033 (PMC2603584; doi:10.1371/journal.pone.0004033)

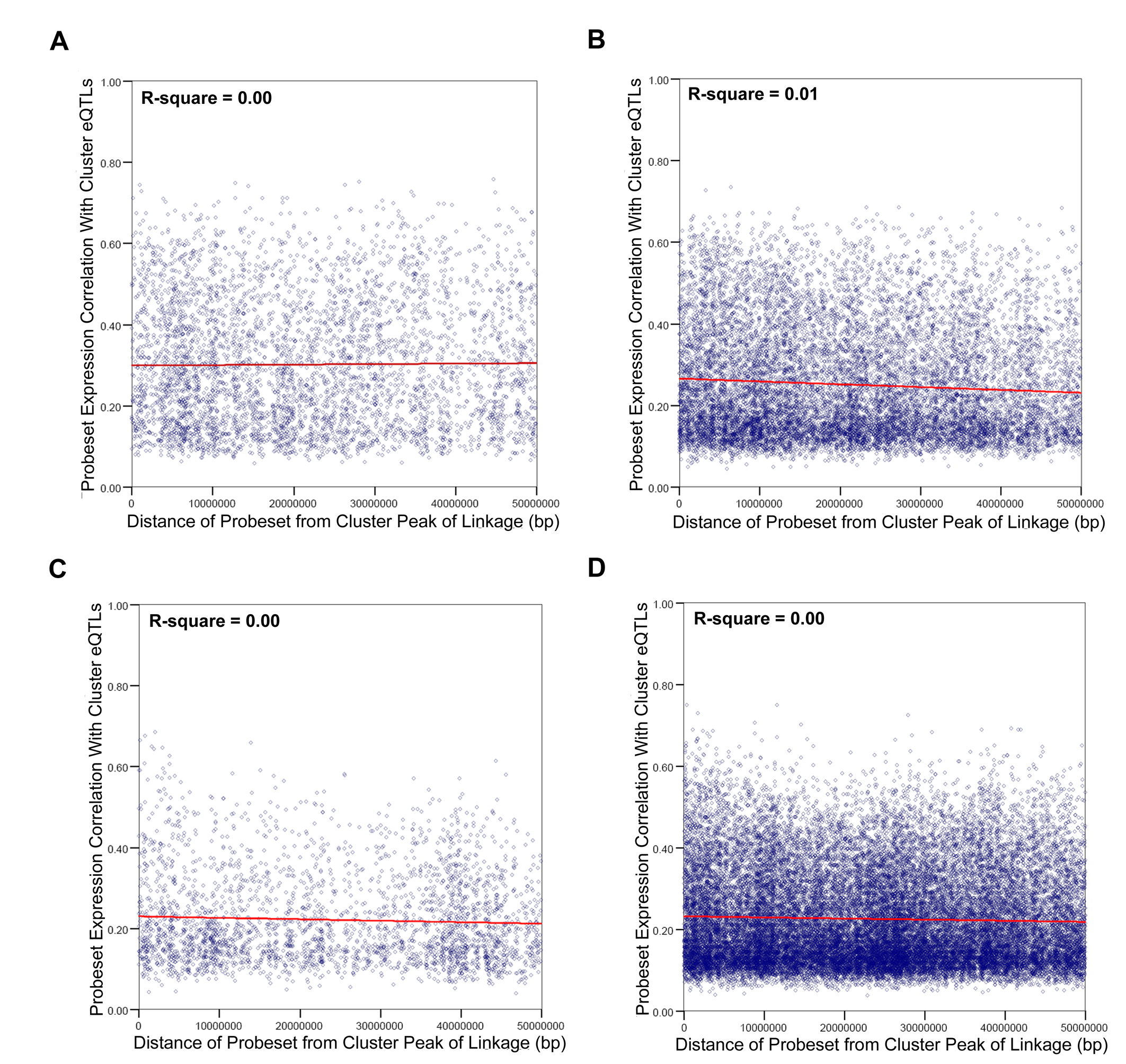

Supplement: Figure S1 — Scatter plots showing correlation of genes underlying cluster trans-eQTLs with all probesets mapped to within 50 Mb of the linkage region, plotted against the physical distance of the probeset from the peak of linkage in a) fat, b) kidney, c) adrenal, d) LV. (6.79 MB TIF) [file pone.0004033.s001.tif]

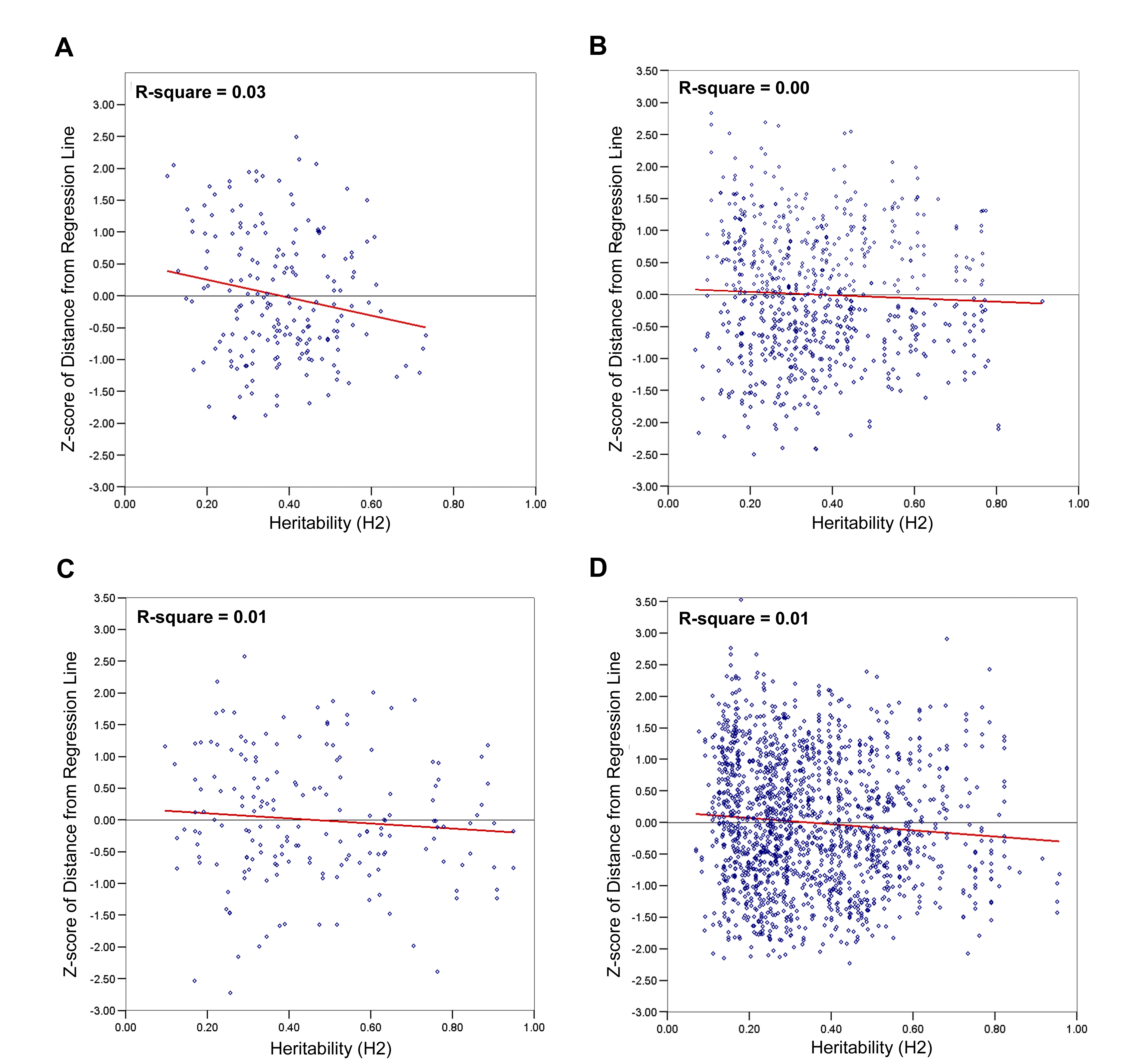

Supplement: Figure S2 — Z-score of cis-eQTL distance from regression line ( Figure 5 ) plotted against probeset heritability in a) fat, b) kidney, c) adrenal or d) LV. (3.24 MB TIF) [file pone.0004033.s002.tif]
