# Supplementary material for: Genome-Wide Co-Expression Analysis in Multiple Tissues
Source: PLoS One. 2008 Dec 29;3(12):e4033. doi: 10.1371/journal.pone.0004033 (PMC2603584; doi:10.1371/journal.pone.0004033)
Supplement: Table S1 — Outcomes of correlation analysis of pairs of cis-eQTL genes (q<0.05), whose peaks of linkage are located more than 50 cM apart. (0.03 MB DOC) [file pone.0004033.s003.doc]

| **Tissue** | **Total Unique Pairs of *cis*-eQTL genes** | **No. Unique Unlinked Pairs of *cis*-eQTL genes (>50cM apart)** | **No. (%) of Significantly Correlated Unlinked Pairs of  *cis*-eQTL Genes** | **No. (%) of Significantly Correlated Unlinked Pairs with Significantly Correlated SDPs** |
| --- | --- | --- | --- | --- |
| Fat | 155,403 | 101,071 | 445 (**0.4**%) | 176 (39.6%) |
| Kidney | 257,403 | 245,045 | 630 (**0.3**%) | 331 (52.5%) |
| Adrenal | 180,901 | 172,242 | 320 (**0.2**%) | 178 (55.6%) |
| LV | 926,841 | 885,896 | 989 (**0.1**%) | 544 (55.0%) |
