# Supplementary material for: Genome-Wide Co-Expression Analysis in Multiple Tissues
Source: PLoS One. 2008 Dec 29;3(12):e4033. doi: 10.1371/journal.pone.0004033 (PMC2603584; doi:10.1371/journal.pone.0004033)
Supplement: Table S2 — Outcomes of correlation analysis of trans-eQTL genes (q<0.05) whose peaks of linkage are located 50 cM or less apart. (0.05 MB DOC) [file pone.0004033.s004.doc]

|  | **Distance Apart of Peaks of Linkage (cM)** | **No. of significantly correlated *trans*-eQTL genes with correlated genotypes at peaks of linkage** | **% of all significantly correlated *trans-*eQTL genes** |
| --- | --- | --- | --- |
| **Fat**  Total Significantly Correlated eQTL genes: **30347_** | ≤ 50 | 13,040 | 43.4 |
|  | ≤ 20 | 12,809 | 42.3 |
|  | ≤ 10 | 12,270 | 40.4 |
|  | ≤ 5 | 11,958 | 39.4 |
|  | ≤ 1 | 11,107 | 36.6 |

|  | **Distance Apart of Peaks of Linkage (cM)** | **No. of significantly correlated *trans*-eQTL genes with correlated genotypes at peaks of linkage** | **% of all significantly correlated *trans-*eQTL genes** |
| --- | --- | --- | --- |
| **Kidney_**  Total Significantly Correlated eQTL genes: **12231_** | ≤ 50 | 4,951 | 41.5 |
|  | ≤ 20 | 4,511 | 37.2 |
|  | ≤ 10 | 4,113 | 33.6 |
|  | ≤ 5 | 3,693 | 30.2 |
|  | ≤ 1 | 2,831 | 23.1 |

|  | **Distance Apart of Peaks of Linkage (cM)** | **No. of significantly correlated *trans*-eQTL genes with correlated genotypes at peaks of linkage** | **% of all significantly correlated *trans-*eQTL genes** |
| --- | --- | --- | --- |
| **Adrenal**  Total Significantly Correlated eQTL genes: **4139_** | ≤ 50 | 2,623 | 63.4 |
|  | ≤ 20 | 2,521 | 60.9 |
|  | ≤ 10 | 2,445 | 59.1 |
|  | ≤ 5 | 2,327 | 56.2 |
|  | ≤ 1 | 1,650 | 39.1 |

|  | **Distance Apart of Peaks of Linkage (cM)** | **No. of significantly correlated *trans*-eQTL genes with correlated genotypes at peaks of linkage** | **% of all significantly correlated *trans-*eQTL genes** |
| --- | --- | --- | --- |
| **Left  Ventricle**  Total Significantly Correlated eQTL genes: **22568** | ≤50 | 11,525 | 52.2 |
|  | ≤20 | 10,859 | 48.1 |
|  | ≤10 | 10,034 | 44.5 |
|  | ≤5 | 9,071 | 40.2 |
|  | ≤1 | 6,727 | 29.8 |
