# Supplementary material for: Genome-Wide Co-Expression Analysis in Multiple Tissues
Source: PLoS One. 2008 Dec 29;3(12):e4033. doi: 10.1371/journal.pone.0004033 (PMC2603584; doi:10.1371/journal.pone.0004033)
Supplement: Table S3 — Outcomes of correlation analysis of expression profiles of genes forming trans-eQTL clusters (0.14 MB DOC) [file pone.0004033.s005.doc]

| **Tissue** | **Marker at *trans*-eQTL cluster peak of linkage** | **No. Transcripts in Cluster** | **No. *cis*-eQTLs within 50Mb of peak of linkage** | **% Significant  *cis*-*trans* expression correlation (*p*<0.01)** | **% Significant  *trans*-*trans* expression correlation (*p*<0.01)** |
| --- | --- | --- | --- | --- | --- |
| Fat | Cacna1s | 33 | 19 | 27.4 | 96.8 |
| Fat | D11Rat7 | 11 | 11 | 23.9 | 85.5 |
| Fat | D12Ntr2 | 19 | 26 | 27.9 | 94.7 |
| Fat | D14Rat52 | 14 | 16 | 21.4 | 100 |
| Fat | D16Cebr204s40 | 20 | 16 | 38.1 | 87.4 |
| Fat | D16Mit1 | 12 | 19 | 47.3 | 87.9 |
| Fat | D17Rat1 | 146 | 17 | 25.8 | 96.8 |
| Fat | D1Rat27 | 19 | 10 | 24.2 | 87.7 |
| Fat | D1Rat7 | 13 | 5 | 50.8 | 97.4 |
| Fat | D4Rat240 | 31 | 19 | 22.4 | 84.7 |
| Fat | D8Utr5 | 21 | 31 | 13.7 | 73.8 |
| Kidney | Abpa | 12 | 13 | 3.2 | 81.8 |
| Kidney | Crabp1 | 18 | 42 | 22.6 | 96.1 |
| Kidney | Cyp45c | 23 | 41 | 14.7 | 83.6 |
| Kidney | D10Cebrp1016s2 | 15 | 48 | 10.8 | 85.7 |
| Kidney | D10Cebrp207s1 | 22 | 45 | 9.6 | 81.8 |
| Kidney | D15Rat29 | 11 | 7 | 24.7 | 72.7 |
| Kidney | D15Rat69 | 57 | 7 | 20.8 | 91.2 |
| Kidney | D16Cebr204s40 | 16 | 19 | 29.9 | 67.5 |
| Kidney | D16Mit2 | 14 | 17 | 46.6 | 79.1 |
| Kidney | D2Cebr11s4 | 13 | 26 | 4.7 | 79.5 |
| Kidney | D3Cebrp1038s1 | 14 | 45 | 2.5 | 74.7 |
| Kidney | D4Mit11 | 13 | 23 | 26.8 | 88.5 |
| Kidney | D4Rat35 | 24 | 22 | 30.9 | 87 |
| Kidney | D4Utr4 | 12 | 24 | 31.3 | 81.8 |
| Kidney | D5Rat174 | 27 | 19 | 26.7 | 90.6 |
| Kidney | D5Rat38 | 10 | 19 | 28.9 | 95.6 |
| Kidney | D8Rat_42 | 17 | 42 | 27.6 | 81.6 |
| Kidney | D8Rat150 | 11 | 41 | 23.7 | 89.1 |
| Kidney | D8Rat21 | 12 | 41 | 12.6 | 90.9 |
| Kidney | D8Utr5 | 16 | 39 | 12.8 | 72.5 |
| Kidney | Igk@ | 49 | 23 | 21.7 | 89.6 |
| Kidney | Scnb2 | 20 | 41 | 33 | 90.5 |
| Kidney | Slc12a1 | 22 | 35 | 24 | 86.1 |
| Adrenal | Abpa | 10 | 16 | 0.6 | 75.6 |
| Adrenal | D11Rat16 | 31 | 11 | 41.3 | 75.5 |
| Adrenal | D15Rat29 | 11 | 5 | 23.6 | 100 |
| Adrenal | D17Rat144 | 47 | 16 | 35.6 | 88.6 |
| Adrenal | D1Utr6 | 11 | 5 | 9.1 | 74.5 |
| Adrenal | D20Mit1 | 11 | 37 | 4.7 | 78.2 |
| Adrenal | D20Rat55 | 16 | 37 | 3 | 80.8 |
| Adrenal | D8Rat56 | 14 | 24 | 29.2 | 83.5 |
| Adrenal | D8Utr3 | 20 | 26 | 28.7 | 85.3 |
| LV | Abpa | 15 | 37 | 1.8 | 73.3 |
| LV | Ckb | 43 | 35 | 23.9 | 79.2 |
| LV | Crabp1 | 165 | 78 | 16.5 | 77.1 |
| LV | Cyp45c | 35 | 78 | 19 | 87.9 |
| LV | D11Cebr11s6 | 11 | 30 | 6.9 | 67.3 |
| LV | D11Mit4 | 10 | 29 | 35.5 | 80 |
| LV | D13Cebr9s2 | 27 | 23 | 39.1 | 86.9 |
| LV | D13Cebr9s3 | 12 | 20 | 53.3 | 72.7 |
| LV | D13Utr6 | 13 | 26 | 30.5 | 66.7 |
| LV | D15Rat123 | 11 | 24 | 4.5 | 74.5 |
| LV | D15Rat29 | 54 | 9 | 48.4 | 99 |
| LV | D15Rat98 | 30 | 13 | 23.8 | 88 |
| LV | D15Ucsf1 | 46 | 13 | 24 | 92.9 |
| LV | D15Utr2 | 51 | 14 | 34.5 | 85.5 |
| LV | D16Cebr204s40 | 15 | 29 | 10.5 | 74.3 |
| LV | D16Mit3 | 13 | 44 | 29.5 | 82.1 |
| LV | D16Rat46 | 28 | 43 | 30.1 | 92.1 |
| LV | D16Rat67 | 26 | 43 | 26.6 | 72.9 |
| LV | D17Mit6 | 20 | 24 | 18.3 | 78.9 |
| LV | D17Rat17 | 10 | 44 | 5.6 | 66.7 |
| LV | D1Cebrp37s18 | 10 | 37 | 6.2 | 60 |
| LV | D20Arb249 | 11 | 28 | 3.9 | 54.5 |
| LV | D3Cebr37s29 | 10 | 64 | 1.9 | 80 |
| LV | D3Mit16 | 14 | 53 | 19.3 | 67 |
| LV | D4Rat140 | 10 | 32 | 16.6 | 82.2 |
| LV | D6Mit10 | 14 | 35 | 24.3 | 93.4 |
| LV | D6Rat79 | 20 | 35 | 23 | 77.9 |
| LV | D6Rat80 | 15 | 11 | 18.1 | 84.8 |
| LV | D8Cebr81s4 | 14 | 66 | 29.4 | 64.8 |
| LV | D8Mgh4 | 10 | 72 | 21.8 | 68.9 |
| LV | D8Mit12 | 77 | 76 | 23.3 | 68.8 |
| LV | D8Rat219 | 21 | 68 | 26.2 | 83.3 |
| LV | D8Rat49 | 11 | 67 | 29.9 | 63.6 |
| LV | D8Utr2 | 10 | 68 | 19.7 | 82.2 |
| LV | D8Utr5 | 22 | 74 | 19.9 | 72.3 |
| LV | Edn3 | 10 | 38 | 23.6 | 68.9 |
| LV | Kcnj1 | 18 | 68 | 24.7 | 82.4 |
| LV | Tpm1 | 23 | 66 | 5.3 | 79.1 |
