# Supplementary material for: Genome-Wide Co-Expression Analysis in Multiple Tissues
Source: PLoS One. 2008 Dec 29;3(12):e4033. doi: 10.1371/journal.pone.0004033 (PMC2603584; doi:10.1371/journal.pone.0004033)
Supplement: Table S4 — Outcomes of correlation of cis-eQTL genes in the ‘window regions’ of trans-eQTL clusters with cluster-forming genes. (0.14 MB DOC) [file pone.0004033.s006.doc]

| **Tissue** | **Marker at *trans*-eQTL cluster peak of linkage** | **No. Transcripts in Cluster** | **No. *cis*-eQTLs within 50Mb of peak of linkage** | **Best-correlated *cis*-eQTL Gene** | **Maximum Correlation Coefficient** | **Average  Correlation**  **Coefficient** |
| --- | --- | --- | --- | --- | --- | --- |
| Fat | Cacna1s | 33 | 19 | 1372839_at | 0.649 | 0.343 |
| Fat | D11Rat7 | 11 | 11 | 1373034_at | 0.601 | 0.353 |
| Fat | D12Ntr2 | 19 | 26 | 1373898_at | 0.647 | 0.353 |
| Fat | D14Rat52 | 14 | 16 | 1374941_at | 0.694 | 0.317 |
| Fat | D16Cebr204s40 | 20 | 16 | 1390435_at | 0.659 | 0.417 |
| Fat | D16Mit1 | 12 | 19 | 1371992_at | 0.674 | 0.441 |
| Fat | D17Rat1 | 146 | 17 | 1369553_at | 0.744 | 0.358 |
| Fat | D1Rat27 | 19 | 10 | 1399157_at | 0.711 | 0.331 |
| Fat | D1Rat7 | 13 | 5 | 1392105_at | 0.657 | 0.446 |
| Fat | D4Rat240 | 31 | 19 | 1372789_at | 0.568 | 0.325 |
| Fat | D8Utr5 | 21 | 31 | 1367808_at | 0.632 | 0.254 |
| Kidney | Abpa | 33 | 13 | 1390104_at | 0.318 | 0.188 |
| Kidney | Crabp1 | 11 | 42 | 1374933_at | 0.671 | 0.301 |
| Kidney | Cyp45c | 19 | 41 | 1374933_at | 0.515 | 0.269 |
| Kidney | D10Cebrp1016s2 | 14 | 48 | 1374050_at | 0.519 | 0.234 |
| Kidney | D10Cebrp207s1 | 20 | 45 | 1367936_at | 0.602 | 0.268 |
| Kidney | D15Rat29 | 12 | 7 | 1388942_at | 0.569 | 0.283 |
| Kidney | D15Rat69 | 146 | 7 | 1373150_at | 0.474 | 0.365 |
| Kidney | D16Cebr204s40 | 19 | 19 | 1390029_at | 0.517 | 0.372 |
| Kidney | D16Mit2 | 13 | 17 | 1387294_at | 0.596 | 0.447 |
| Kidney | D2Cebr11s4 | 31 | 26 | 1367838_at | 0.435 | 0.211 |
| Kidney | D3Cebrp1038s1 | 21 | 45 | 1375068_at | 0.382 | 0.177 |
| Kidney | D4Mit11 | 13 | 23 | 1388076_at | 0.584 | 0.338 |
| Kidney | D4Rat35 | 24 | 22 | 1388076_at | 0.605 | 0.353 |
| Kidney | D4Utr4 | 12 | 24 | 1368010_at | 0.592 | 0.339 |
| Kidney | D5Rat174 | 27 | 19 | 1370825_a_at | 0.625 | 0.320 |
| Kidney | D5Rat38 | 10 | 19 | 1371960_at | 0.591 | 0.334 |
| Kidney | D8Rat_42 | 17 | 42 | 1374933_at | 0.609 | 0.317 |
| Kidney | D8Rat150 | 11 | 41 | 1372403_at | 0.604 | 0.312 |
| Kidney | D8Rat21 | 12 | 41 | 1370067_at | 0.581 | 0.242 |
| Kidney | D8Utr5 | 16 | 39 | 1371689_at | 0.510 | 0.266 |
| Kidney | Igk@ | 49 | 23 | 1388076_at | 0.647 | 0.316 |
| Kidney | Scnb2 | 20 | 41 | 1374933_at | 0.663 | 0.337 |
| Kidney | Slc12a1 | 22 | 35 | 1370156_at | 0.624 | 0.324 |
| Adrenal | Abpa | 10 | 16 | 1367778_at | 0.233 | 0.155 |
| Adrenal | D11Rat16 | 31 | 11 | 1370947_at | 0.647 | 0.396 |
| Adrenal | D15Rat29 | 11 | 5 | 1388942_at | 0.576 | 0.282 |
| Adrenal | D17Rat144 | 47 | 16 | 1368225_at | 0.684 | 0.358 |
| Adrenal | D1Utr6 | 11 | 5 | 1374130_at | 0.470 | 0.205 |
| Adrenal | D20Mit1 | 11 | 37 | 1370948_a_at | 0.487 | 0.176 |
| Adrenal | D20Rat55 | 16 | 37 | 1370948_a_at | 0.576 | 0.166 |
| Adrenal | D8Rat56 | 14 | 24 | 1398460_at | 0.626 | 0.311 |
| Adrenal | D8Utr3 | 20 | 26 | 1372805_at | 0.666 | 0.323 |
| LV | Abpa | 15 | 37 | 1379655_at | 0.371 | 0.168 |
| LV | Ckb | 43 | 35 | 1377541_at | 0.592 | 0.290 |
| LV | Crabp1 | 165 | 78 | 1392020_at | 0.620 | 0.287 |
| LV | Cyp45c | 35 | 78 | 1372103_at | 0.633 | 0.299 |
| LV | D11Cebr11s6 | 11 | 30 | 1377655_at | 0.445 | 0.205 |
| LV | D11Mit4 | 10 | 29 | 1380533_at | 0.629 | 0.386 |
| LV | D13Cebr9s2 | 27 | 23 | 1382394_at | 0.588 | 0.429 |
| LV | D13Cebr9s3 | 12 | 20 | 1393023_at | 0.668 | 0.462 |
| LV | D13Utr6 | 13 | 26 | 1379286_at | 0.592 | 0.388 |
| LV | D15Rat123 | 15 | 24 | 1383279_at | 0.407 | 0.170 |
| LV | D15Rat29 | 43 | 9 | 1385378_at | 0.731 | 0.404 |
| LV | D15Rat98 | 165 | 13 | 1393678_at | 0.681 | 0.309 |
| LV | D15Ucsf1 | 35 | 13 | 1393678_at | 0.671 | 0.336 |
| LV | D15Utr2 | 11 | 14 | 1374645_at | 0.652 | 0.395 |
| LV | D16Cebr204s40 | 10 | 29 | 1386773_at | 0.523 | 0.275 |
| LV | D16Mit3 | 27 | 44 | 1370939_at | 0.667 | 0.333 |
| LV | D16Rat46 | 12 | 43 | 1374193_at | 0.664 | 0.345 |
| LV | D16Rat67 | 13 | 43 | 1370939_at | 0.631 | 0.328 |
| LV | D17Mit6 | 15 | 24 | 1380476_at | 0.582 | 0.316 |
| LV | D17Rat17 | 43 | 44 | 1380833_at | 0.613 | 0.195 |
| LV | D1Cebrp37s18 | 165 | 37 | 1389152_at | 0.457 | 0.211 |
| LV | D20Arb249 | 35 | 28 | 1369412_a_at | 0.446 | 0.162 |
| LV | D3Cebr37s29 | 11 | 64 | 1375517_at | 0.415 | 0.186 |
| LV | D3Mit16 | 10 | 53 | 1392077_at | 0.553 | 0.311 |
| LV | D4Rat140 | 27 | 32 | 1374505_at | 0.570 | 0.216 |
| LV | D6Mit10 | 12 | 35 | 1392212_at | 0.652 | 0.281 |
| LV | D6Rat79 | 13 | 35 | 1368831_at | 0.568 | 0.296 |
| LV | D6Rat80 | 15 | 11 | 1384149_at | 0.530 | 0.288 |
| LV | D8Cebr81s4 | 43 | 66 | 1377061_at | 0.660 | 0.325 |
| LV | D8Mgh4 | 165 | 72 | 1391235_at | 0.560 | 0.328 |
| LV | D8Mit12 | 35 | 76 | 1372103_at | 0.650 | 0.316 |
| LV | D8Rat219 | 11 | 68 | 1375988_at | 0.681 | 0.317 |
| LV | D8Rat49 | 10 | 67 | 1377061_at | 0.691 | 0.327 |
| LV | D8Utr2 | 27 | 68 | 1394154_at | 0.598 | 0.303 |
| LV | D8Utr5 | 12 | 74 | 1397569_at | 0.635 | 0.289 |
| LV | Edn3 | 13 | 38 | 1374921_at | 0.643 | 0.290 |
| LV | Kcnj1 | 12 | 68 | 1375988_at | 0.672 | 0.311 |
| LV | Tpm1 | 13 | 66 | 1378080_at | 0.470 | 0.209 |
