# Supplementary material for: Genome-Wide Co-Expression Analysis in Multiple Tissues
Source: PLoS One. 2008 Dec 29;3(12):e4033. doi: 10.1371/journal.pone.0004033 (PMC2603584; doi:10.1371/journal.pone.0004033)
Supplement: Table S5 — cis-eQTL genes located within the window region of a trans-eQTL cluster positively deviating (Z>2) from regression of cluster-averaged correlation coefficient against distance of cis-eQTL from linkage region ( Figure 5 ). (0.08 MB DOC) [file pone.0004033.s007.doc]

| **Tissue** | **Marker at *trans*-eQTL cluster peak of linkage** | ***cis*-eQTL Transcript** | **Average correlation with cluster** | **cM Distance from  peak of linkage** | **Z-score** |
| --- | --- | --- | --- | --- | --- |
| LV | D15Utr2 | 1374645_at | 0.652 | 26.3 | 3.536 |
| LV | D8Utr5 | 1397569_at | 0.635 | 23.4 | 2.916 |
| Kidney | D4Utr4 | 1368908_at | 0.538 | 42.6 | 2.844 |
| LV | D16Rat46 | 1383095_at | 0.577 | 22.3 | 2.771 |
| Kidney | D4Mit11 | 1371635_at | 0.570 | 12.7 | 2.700 |
| LV | D16Mit3 | 1387762_s_at | 0.638 | 11.8 | 2.671 |
| LV | D16Mit3 | 1383095_at | 0.554 | 20.0 | 2.663 |
| Kidney | D4Rat35 | 1368908_at | 0.578 | 14.9 | 2.661 |
| Kidney | Slc12a1 | 1389290_at | 0.457 | 38.9 | 2.645 |
| Adrenal | D8Utr3 | 1376602_a_at | 0.425 | 64.5 | 2.579 |
| Kidney | D8Utr5 | 1371689_at | 0.510 | 24.7 | 2.555 |
| Kidney | Crabp1 | 1374933_at | 0.671 | 11.6 | 2.529 |
| Kidney | Slc12a1 | 1371772_at | 0.534 | 33.0 | 2.528 |
| Fat | Cacna1s | 1372839_at | 0.649 | 54.5 | 2.494 |
| LV | D16Rat67 | 1383095_at | 0.517 | 27.1 | 2.494 |
| LV | D13Cebr9s3 | 1393023_at | 0.668 | 2.8 | 2.428 |
| LV | D15Rat29 | 1385378_at | 0.731 | 1.8 | 2.392 |
| LV | D13Cebr9s3 | 1378831_at | 0.612 | 14.3 | 2.379 |
| LV | D16Rat46 | 1393662_at | 0.548 | 19.1 | 2.343 |
| LV | D8Mgh4 | 1391235_at | 0.560 | 5.5 | 2.321 |
| LV | D15Ucsf1 | 1393678_at | 0.671 | 10.0 | 2.318 |
| LV | D8Utr5 | 1382105_at | 0.551 | 23.4 | 2.310 |
| LV | D16Rat67 | 1387762_s_at | 0.572 | 18.9 | 2.299 |
| Kidney | Slc12a1 | 1371611_at | 0.503 | 33.1 | 2.293 |
| LV | D15Rat98 | 1393678_at | 0.681 | 8.6 | 2.290 |
| LV | D8Mgh4 | 1383626_at | 0.538 | 34.7 | 2.288 |
| LV | D13Utr6 | 1391613_at | 0.556 | 17.6 | 2.279 |
| LV | D8Mgh4 | 1393079_at | 0.479 | 37.7 | 2.235 |
| Kidney | D4Mit11 | 1368908_at | 0.524 | 10.8 | 2.227 |
| LV | D16Rat46 | 1374193_at | 0.664 | 18.0 | 2.201 |
| LV | D16Rat67 | 1393662_at | 0.506 | 23.9 | 2.200 |
| Kidney | Igk@ | 1371635_at | 0.516 | 15.2 | 2.200 |
| Adrenal | D20Mit1 | 1377136_at | 0.432 | 66.6 | 2.184 |
| LV | D8Mgh4 | 1394154_at | 0.464 | 38.3 | 2.164 |
| Fat | D8Utr5 | 1382105_at | 0.586 | 23.4 | 2.144 |
| LV | D16Mit3 | 1393662_at | 0.511 | 16.8 | 2.136 |
| Kidney | D4Utr4 | 1373539_at | 0.432 | 43.7 | 2.135 |
| LV | D13Cebr9s2 | 1382394_at | 0.588 | 5.3 | 2.106 |
| LV | D13Utr6 | 1383264_at | 0.553 | 13.5 | 2.106 |
| LV | D8Mgh4 | 1373055_at | 0.493 | 33.7 | 2.106 |
| LV | D8Rat49 | 1377061_at | 0.691 | 1.3 | 2.089 |
| Kidney | D8Rat_42 | 1372403_at | 0.609 | 3.5 | 2.077 |
| LV | D8Rat219 | 1375988_at | 0.681 | 1.5 | 2.076 |
| Fat | Cacna1s | 1374505_at | 0.592 | 51.0 | 2.070 |
| LV | D15Utr2 | 1393678_at | 0.621 | 11.5 | 2.068 |
| Fat | D17Rat1 | 1369553_at | 0.744 | 1.2 | 2.052 |
| LV | D8Mgh4 | 1397936_at | 0.495 | 32.3 | 2.049 |
| LV | D13Cebr9s3 | 1387074_at | 0.496 | 31.7 | 2.028 |
| LV | Kcnj1 | 1375988_at | 0.672 | 4.7 | 2.027 |
| Adrenal | D17Rat144 | 1368225_at | 0.684 | 1.9 | 2.015 |
| LV | D13Cebr9s2 | 1392247_at | 0.508 | 19.6 | 2.012 |
| Kidney | Scnb2 | 1374933_at | 0.663 | 2.8 | 2.010 |
| Kidney | D8Utr5 | 1382105_at | 0.460 | 23.4 | 2.001 |
