# Supplementary material for: Genome-Wide Co-Expression Analysis in Multiple Tissues
Source: PLoS One. 2008 Dec 29;3(12):e4033. doi: 10.1371/journal.pone.0004033 (PMC2603584; doi:10.1371/journal.pone.0004033)
Supplement: Table S6 — Supplementary information on functional enrichment analysis of large (>30 transcripts) trans-eQTL clusters. (0.04 MB DOC) [file pone.0004033.s008.doc]

| **Tissue** | **Marker at Cluster Peak of Linkage** | **No. Transcripts in Cluster** | **No. Annotated Transcripts** | **No. KEGG Pathways** |
| --- | --- | --- | --- | --- |
| LV | D15Rat98 | 30 | 17 | 0 |
| Adrenal | D11Rat16 | 31 | 25 | 0 |
| Fat | D4Rat240 | 31 | 28 | 4 |
| Fat | Cacna1s | 33 | 25 | 1 |
| LV | Cyp45c | 35 | 20 | 0 |
| LV | Ckb | 43 | 26 | 0 |
| LV | D15Ucsf1 | 46 | 32 | 6 |
| Adrenal | D17Rat144 | 47 | 35 | 1 |
| Kidney | Igk@ | 49 | 33 | 0 |
| LV | D15Utr2 | 51 | 33 | 0 |
| LV | D15Rat29 | 54 | 40 | 1 |
| Kidney | D15Rat69 | 57 | 47 | 0 |
| LV | D8Mit12 | 77 | 44 | 7 |
| Fat | D17Rat1 | 146 | 116 | 3 |
| LV | Crabp1 | 165 | 101 | 0 |
